# Diagnostic accuracy of ultrasound for the assessment of Baker’s cysts: a meta-analysis

**DOI:** 10.1186/s13018-022-03430-9

**Published:** 2022-12-12

**Authors:** Ke Liu, Xiaoxiao Li, Qianlin Weng, Guanghua Lei, Ting Jiang

**Affiliations:** 1 Department of Orthopaedics, Xiangya Hospital, Central South University, Changsha, China; 2 Hunan Key Laboratory of Joint Degeneration and Injury, Xiangya Hospital, Central South University, Changsha, China; 3 National Clinical Research Center for Geriatric Disorders, Xiangya Hospital, Central South University, Changsha, China; 4 Department of Ultrasonography, Xiangya Hospital, Central South University, 87 Xiangya Road, Changsha, 410008 Hunan China

**Keywords:** Backer’s cyst, Ultrasound, Diagnostic accuracy, MRI, Pathology

## Abstract

**Background:**

Baker’s cyst is the most common cystic disease of the knee, and a fast and accurate diagnosis of Baker’s cyst is essential for a better management. Ultrasound is a rapid, portable, widely available, inexpensive and noninvasive imaging modality. However, the diagnostic accuracy of ultrasound on Baker’s cyst still remains undetermined. We conducted the first meta-analysis to comprehensively assess the accuracy of ultrasound for the detection of Baker’s cyst.

**Methods:**

PubMed, Embase and Web of Science were searched from inception to July 14, 2022, without language restrictions. Studies providing cross-tabulations of ultrasound versus pathology (gold standard) or MRI (standard imaging technique) for diagnosis of Baker’s cyst were included. Indicators for the diagnostic accuracy of ultrasound, including sensitivity, specificity and area under the curve, were calculated using a bivariate model. Sensitivity analysis was conducted to evaluate the heterogeneity and robustness of the results.

**Results:**

A total of 13 studies with 1,011 subjects (mean age 32.2 years; men 53.5%) met the inclusion criteria. The pooled sensitivity, specificity and area under the curve of ultrasound for diagnosis of Baker’s cyst, compared with pathology, were 0.97 (95% confidence intervals: 0.73–1.00), 1.00 (0.98–1.00) and 1.00 (0.99–1.00), respectively. The pooled estimates of ultrasound versus MRI were 0.94 (0.87–0.98) for sensitivity, 1.00 (0.83–1.00) for specificity and 0.97 (0.95–0.98) for area under the curve. Sensitivity analysis did not change the results materially.

**Conclusion:**

Ultrasound shows excellent diagnostic accuracy for the assessment of Baker’s cyst and provides similar diagnostic information (absent or present) compared to MRI. Because of its advantages of low cost, portability and accessibility, ultrasound is likely to be a choice of imaging technique for screening Baker’s cyst in clinical and population settings as well as in follow-ups.

**Supplementary Information:**

The online version contains supplementary material available at 10.1186/s13018-022-03430-9.

## Background

Baker’s cyst (BC), also known as popliteal cyst, is the most common cystic disease of the knee, which affects approximately 11.7% of the middle-aged and elderly population [1–3]. The clinical manifestations of BC include knee pain, localized swelling or mass, limited range of motion and even peripheral neuropathy and ischemia [4–7]. BC has demonstrated a longitudinal association with the radiological and clinical progression of osteoarthritis [8] and may therefore be considered a predictor of osteoarthritis [9] and a modifiable treatment target [10]. Early diagnosis of BC may prevent further complications connected with the direct compression or ruptures to the surrounding tissues [11]. However, it may be a challenging task for physicians to differentiate BC from other musculoskeletal disorders in or near the popliteal fossa, such as synovial and bone tumor, meniscal cyst and ganglion cyst, which share similar symptoms and signs [12]. Therefore, a tool for fast and accurate diagnosis of BC is needed and could contribute to better management among patients with BC.

Pathological diagnosis is the gold standard for the diagnosis of BC. However, it requires an invasive procedure, such as biopsy or surgical excision. In clinical practice, diagnosis of BC is commonly confirmed by noninvasive imaging, e.g., magnetic resonance imaging (MRI), especially for small and asymptomatic BC [12, 13]. MRI has also been considered the reference standard of imaging in diagnosis of BC [1, 14, 15]. Nevertheless, owing to its limited availability and long examination time, MRI is rarely used as the initial evaluation method for patients with BC and is generally applied as a diagnostic option when a patient experiences persistent symptom despite adequate conservative treatment or when surgery is considered [16, 17]. Otherwise, BC is, for the most part, asymptomatic and necessitates no treatment.

Ultrasonography is a rapid, portable, widely available, inexpensive and noninvasive imaging modality that has been proposed as a promising imaging tool for the assessment of BC [15, 18, 19]. However, a general view is that ultrasound is not as accurate as MRI in diagnosing BC (absent or present) [19, 20]. Currently, there are no published systematic investigations regarding the diagnostic accuracy of ultrasound for BC. To fill this knowledge gap, we conducted the first ever meta-analysis to determine the diagnostic accuracy of ultrasound for BC compared to both surgical pathology and MRI.

## Methods

### Protocol

This study was conducted in accordance with the Preferred Reporting Items for Systematic Reviews and Meta-analyses (PRISMA) reporting guidelines [21]. A predefined protocol of this study was registered with PROSPERO (ID = CRD42022343307).

### Literature search

PubMed, Embase and Web of Science databases were systematically searched from inception up to July 14, 2022. Keywords or Medical Subject Headings terms were properly defined with necessary adaptions to all the search terms for the purpose of satisfying the specific search and syntax rules of three databases (see Additional file 1: Data 1 for the complete electronic search strategy). The reference lists of included studies were searched manually to identify additional studies. Two reviewers (KL and XL) screened the data sources independently, and disagreement, if any, between them was resolved by consulting a third reviewer (TJ).

### Inclusion/exclusion criteria

Articles meeting the following criteria were included: (1) studies using both ultrasound and pathology or both ultrasound and MRI to assess BC with any diagnostic criteria; (2) studies providing a table of true-positive, false-positive, true-negative and false-negative counts, which can be used to evaluate the diagnostic accuracy of ultrasound based on pathology and MRI, respectively, as the reference standard; and (3) no language and study design restrictions.

The exclusion criteria were as follows: (1) studies involving cadaver subjects or non-human research; (2) duplicate publications (if multiple published studies were retrieved from the same database, the largest or the most complete one was included); (3) reviews; or (4) abstract or title publication only.

### Quality assessment

The risk of bias was evaluated by two reviewers (KL and XL) with the tool of quality assessment for diagnostic accuracy studies-2 (QUADAS-2) [22]. In the case of disagreement, consensus would be reached by consultation. The QUADAS-2 tool mainly concerns the bias and applicability of the study results, including patient selection, index test, reference standard and study flow and timing.

### Data extraction

Two investigators (KL and XL) performed data extraction independently using a standardized form. Any disagreements would be resolved by consulting an authoritative third reviewer (TJ). Data were collected on the study characteristics (year of publication, country and type of study), patient characteristics, features of ultrasound, MRI and pathology, pathological findings and a cutoff value of BC and the 2 × 2 diagnostic table. Ultrasound was considered for the index test by taking pathology and MRI as the reference standard, respectively. The diagnostic table was extracted at the joint level.

### Statistical analysis

To assess the diagnostic efficacy of ultrasound, a bivariate model was used to calculate the diagnostic odds ratio (DOR), the summary receiver operating characteristic (SROC), area under the curve (AUC), as well as the pooled sensitivity, specificity, positive likelihood ratio (PLR) and negative likelihood ratio (NLR), along with their 95% confidence intervals (95% CI) [23]. *P *values less than 0.05 were considered statistically significant. Heterogeneity was assessed using the *I*^2^ test (0–40%: low; 30–60%: moderate; 50–90%: substantial; and 75–100%: considerable) [24]. The sensitivity analysis was performed by excluding small-sample studies or studies involving a high risk of bias to evaluate the heterogeneity and robustness.

All statistical analyses were performed with STATA (V.15.0, Stata, College Station, Texas, USA) and/or Review Manager 5.4 (RevMan 5.4, The Cochrane Collaboration, Oxford, UK).

## Results

### Study characteristics

**Fig. 1:**
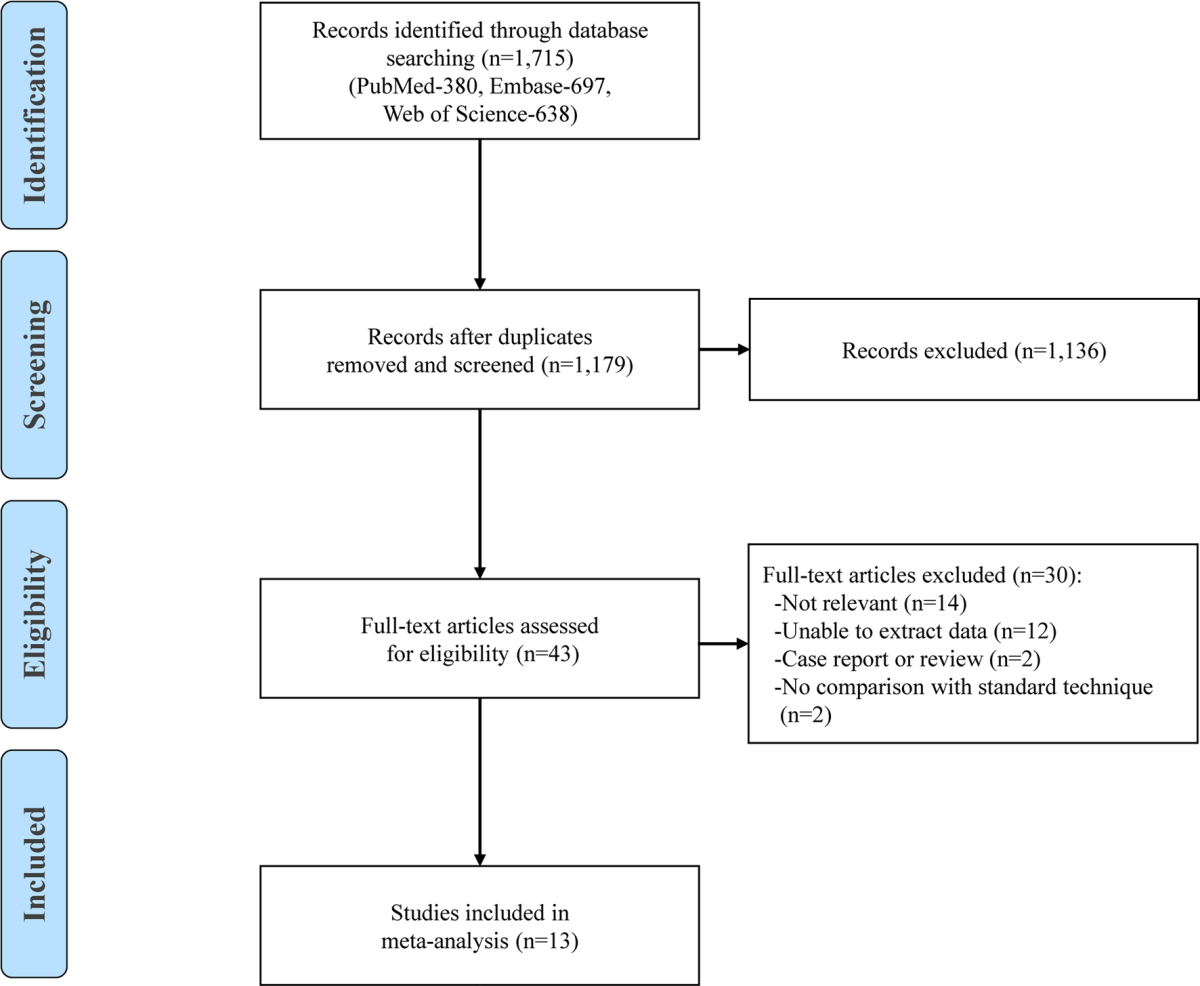
Summary of database search and study selection according to PRISMA flow diagram

The results of database search are summarized in the PRISMA flowchart as shown in Fig. 1. Of the 1715 reports screened, 13 studies (four studies reporting ultrasound versus pathology and nine studies reporting ultrasound versus MRI) met the selection criteria and were included in this meta-analysis, covering a total of 1011 patients with 1033 knees [15, 18–20, 25–33]. The mean age of patients was 32.2 years, and 53.5% were men. The details of the study characteristics are presented in Table 1. Of the included studies, seven were conducted in developed countries and six in developing countries. All the BCs were recorded using a dichotomous scale (either present or absent) based on information retrieved from the original studies. Additional file 1: Figs. 1 and 2 demonstrate the methodological quality of each study.

**Table 1:** Characteristics of included studies

First author	Year	Country	Patients/knees, n	Females, n	Age, mean years	Reference test
Singh	2021	India	103/103	24	32.9	MRI
Sadeghian	2018	Iran	83/83	NA	51.3	MRI
Makarova	2018	Russia	19/19	12	22.6	MRI
Singh	2016	India	50/50	NA	NA	MRI
Wu	2013	China	397/397	173	29.1	Pathology
An	2011	Korea	154/154	89	44.7	Pathology
Neubauer	2011	Germany	16/24	NA	NA	MRI
Yucesoy	2011	Turkey	30/30	25	55.9	MRI
Ward	2001	America	36/36	17	46.0	MRI
El-Miedany	2001	Egypt	38/38	25	8.0	MRI
Abiezzi	1995	America	44/44	17	NA	Pathology
Ostergaard	1995	Denmark	20/34	NA	29.0	MRI
Toolanen	1988	Sweden	21/21	NA	40.0	Pathology

US, ultrasound; MRI, magnetic resonance imaging; and NA, not available

**Fig. 2:**
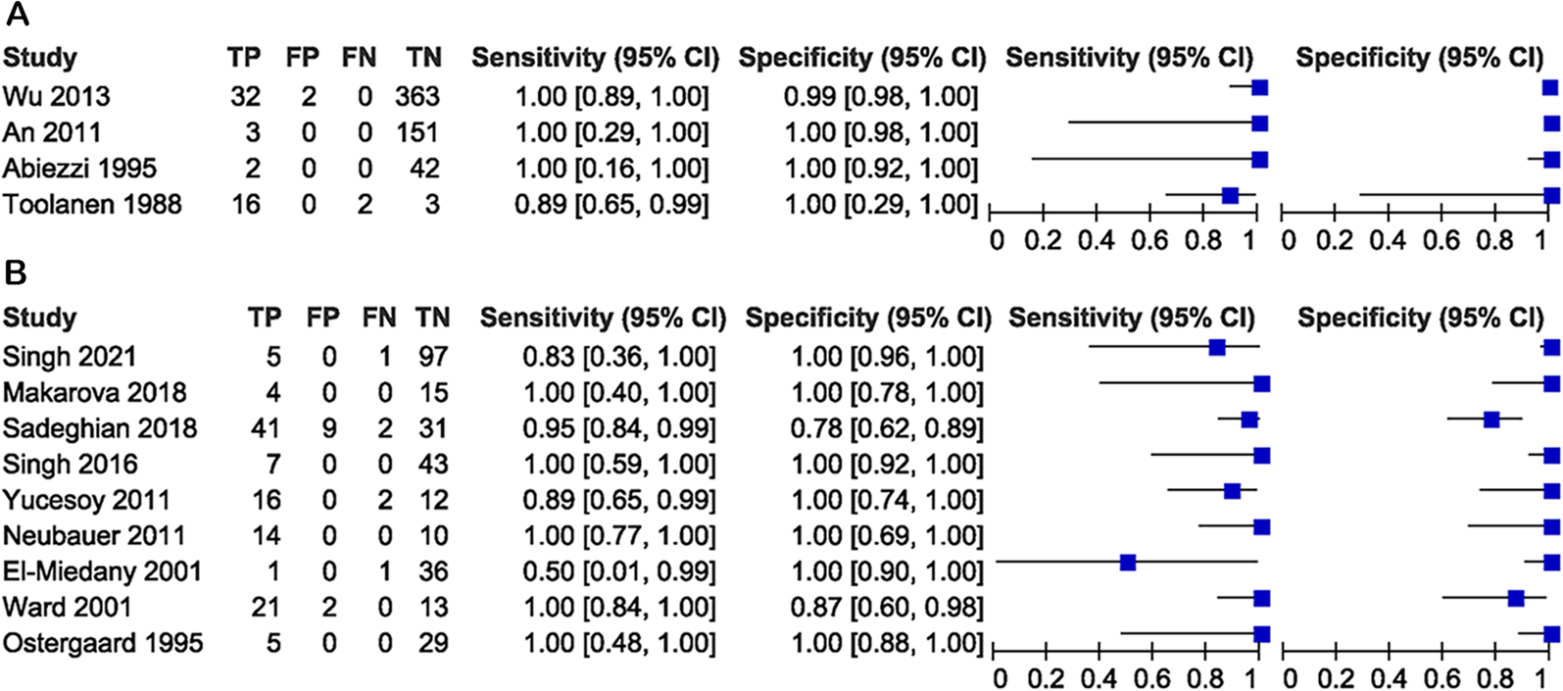
The paired forest plots for the diagnostic accuracy of ultrasound. **A** The reference test was pathology; **B** the reference test was MRI. TP, true positive; FP, false positive; FN, false negative; TN, true negative; and CI, confidence intervals

### Diagnostic value of ultrasound compared with pathology

**Fig. 3:**
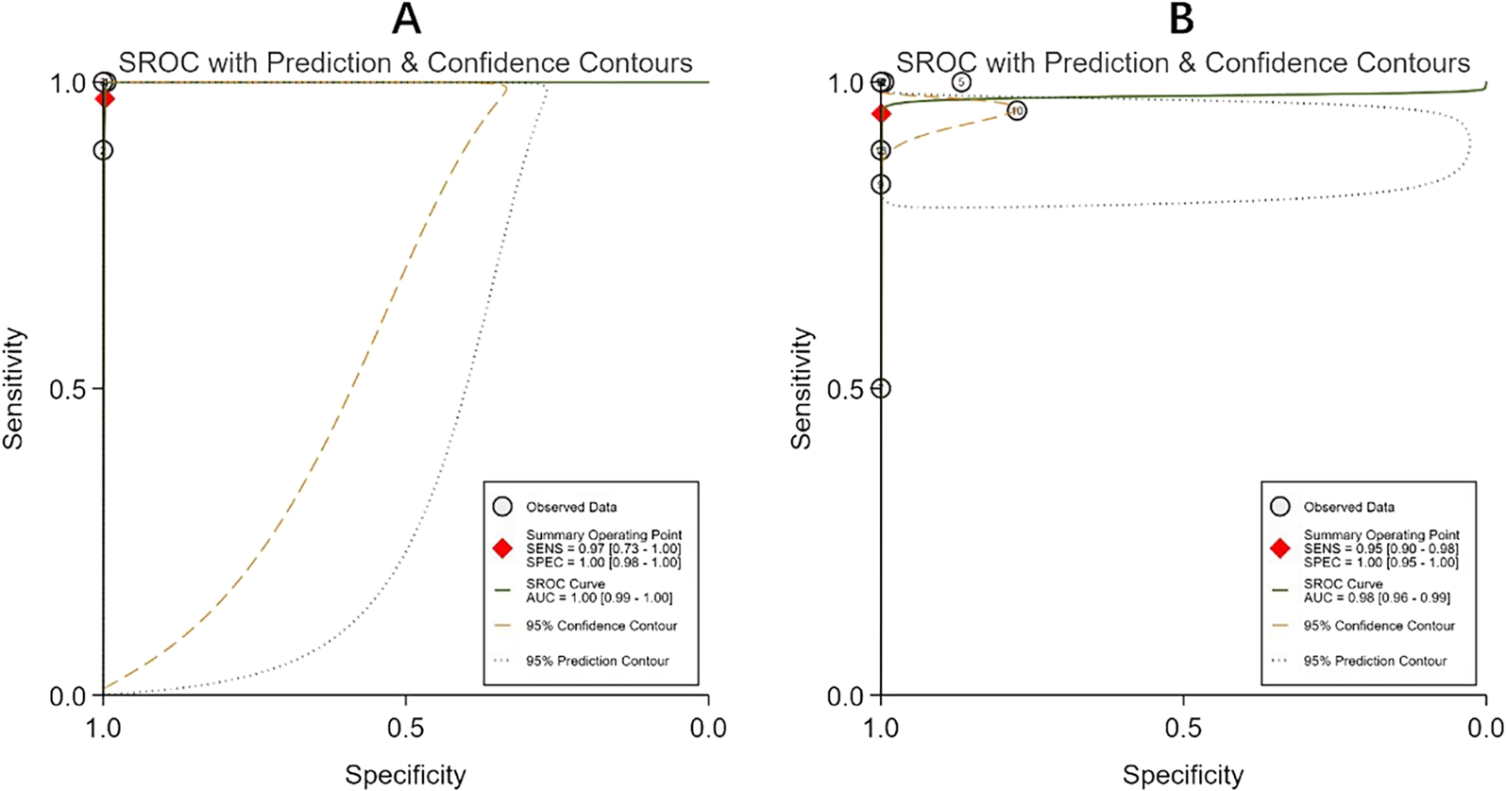
Summary receiver operating characteristic (SROC) curves of ultrasound. **A** The reference test was pathology; **B** the reference test was MRI. SENS, sensitivity; SPEC, specificity; and AUC, area under the curve

Four studies with a total of 616 patients reported the diagnostic accuracy of ultrasound versus pathology for BCs and yielded an AUC of 1.00 (95% CI 0.99–1.00, *I*^2^ = 0) and a DOR of 12,335 (95% CI 546–278,517, *I*^2^ = 99.97) (Table 2; Figs. 2, 3). The overall sensitivity, specificity, PLR and NLR were 0.97 (95% CI 0.73–1.00, *I*^2^ = 29.68), 1.00 (95% CI 0.98–1.00, *I*^2^ = 0), 335.47 (95% CI 42.60–2641.68, *I*^2^ = 34.67) and 0.03 (95% CI 0.00–0.34, *I*^2^ = 62.01), respectively (Table 2; Fig. 2). Because the diagnostic accuracy of ultrasound versus pathology was only reported by four studies and a low degree of heterogeneity was observed, the sensitivity analysis was not performed.

**Table 2:** Summary of diagnostic accuracy results of ultrasound versus reference test

Reference test	AUC (95% CI)	Sensitivity (95% CI)	Specificity (95% CI)	PLR (95% CI)	NLR (95% CI)	DOR (95% CI)
Pathology	1.00 (0.99, 1.00)	0.97 (0.73, 1.00)	1.00 (0.98, 1.00)	335.47 (42.60, 2641.68)	0.03 (0.00, 0.34)	12,335 (546, 278,517)
MRI	0.97 (0.95, 0.98)	0.94 (0.87, 0.98)	1.00 (0.83, 1.00)	483.38 (4.55, 51,407.24)	0.06 (0.03, 0.13)	8318 (80, 866,026)

AUC, area under the curve; PLR, positive likelihood ratio; NLR, negative likelihood ratio; DOR, diagnostic odds ratio; CI, confidence intervals; and MRI, magnetic resonance imaging

### Diagnostic value of ultrasound compared with MRI

Nine studies with a total of 395 patients reported the diagnosis accuracy of ultrasound versus MRI for BCs and yielded an AUC of 0.97 (95% CI 0.95–0.98, *I*^2^ = 87) and a DOR of 8,318 (95% CI 80–866,026, *I*^2^ = 100) (Table 2; Figs. 2, 3). The overall sensitivity was 0.94 (95% CI 0.87–0.98, *I*^2^ = 86.66), specificity was 1.00 (95% CI 0.83–1.00, *I*^2^ = 91.23), PLR was 483.38 (95% CI 4.55–51,407.24, *I*^2^ = 84.31) and NLR was 0.06 (95% CI 0.03–0.13, *I*^2^ = 89.48), respectively (Table 2; Fig. 2). In sensitivity analysis, the pooled results of diagnostic accuracy were found to be robust (AUC 0.97 95% CI 0.83–0.97) after removing two small-sample studies (number of patients < 30) [18, 26], but the level of heterogeneity remained high. After removing four studies involving a high risk of bias [19, 20, 25, 31], the pooled results of diagnostic accuracy were still robust (AUC 0.98 95% CI 0.76–1.00) and the heterogeneity of AUC was significantly reduced, with the I^2^ index decreasing from 87 to 22%. The study quality assessed by QUADAS-2 may influence the degree of heterogeneity among the included studies.

## Discussion

This meta-analysis comprehensively assessed the diagnostic performance of ultrasound versus reference standard (i.e., pathology and MRI) for BC. Our results suggest that ultrasound shows excellent diagnostic accuracy for BC as compared with pathology. Moreover, the use of ultrasound provides similar diagnostic information (absent or present) compared to MRI for the evaluation of BC.

Multiple imaging approaches have been used in the diagnosis and assessment of BC, among which MRI is considered to be the reference standard. However, the limited availability and high cost of MRI decrease its application value for diagnosing BC in clinical practice, especially for quickly assessing potential BC or screening asymptomatic patients [16, 17]. In contrast, ultrasound is widely available and low cost and has shown excellent diagnostic accuracy. Anechoic or hypoechoic fluid between the semimembranosus and the medial head of the gastrocnemius could be accurately detected even when the transverse diameter is less than 4 mm [34]. However, ultrasound has its shortcomings. It is deemed an operator-dependent technique and does require consideration of appropriate training and quality assessment. However, Oo et al. showed that the pooled kappa of a binary score was almost perfect for BCs (inter-rater reliability, 0.92 [0.83–1.00]) [35]. Moreover, ultrasound is not sensitive to intra-articular lesions, and therefore, further imaging is needed to confirm the presence of an associated internal derangement [15]. For instance, MRI can provide additional information during the development of surgical plans when detailed evaluation of deep knee structures and the overall profile of the joint are required.

In addition to the dichotomous diagnosis of BC, other additional features of BC, e.g., the opening (the communication between the gastrocnemio-semimembranosus bursa and the knee joint capsule) and volume of BC, have been accurately detected by ultrasound as reported in previous studies. Assessment of the opening of BC was often carried out by MRI preoperatively, and the results could guide the formulation of clinical surgical plans [14, 36]. Two previous studies suggested that ultrasound was able to diagnose the opening of BC [15, 18], but no detailed investigation has been reported yet on the diagnostic accuracy of ultrasound versus MRI on this topic. In addition, ultrasound was also thought to be able to estimate the volume of BC [18], which was closely related to the development of symptoms among patients with BC [37–39]. However, owing to the lack of published data, this additional information about BCs could not be included in our meta-analysis. Further studies with a focus on the diagnostic accuracy of ultrasound on features of BC are therefore needed.

Since ultrasound could provide highly efficient and accurate information in diagnosing BC, the use of ultrasound may facilitate clinical management and decision-making with reduced cost and time consumption. In view of advantages and disadvantages of each imaging modality, the choice of which one to assess BC depends on the requirement of the referring clinicians and researchers. If they want to know whether a BC exists in a patient with a well-defined intra-articular disorder, such as osteoarthritis or rheumatoid arthritis, or in participants of population screening and cohort follow-ups, ultrasound is the technique of choice. If they already know that a BC is present, ultrasound can be used to assess complications, such as rupture and compression. In addition, ultrasound can also be performed as a real-time guide for biopsies, fluid aspiration and injection of medication, which may contribute to individualized treatment [40].

As a major strength, our study provided novel evidence that ultrasound could present similar diagnostic information (absent or present) for BC compared to MRI, with accumulation of evidence from an enlarged sample. Moreover, we conducted a systematic literature search regardless of language or reporting types across three different databases in order to identify available published studies as comprehensively as possible. Lastly, the results of sensitivity analysis supported the robustness of most findings.

However, this meta-analysis has several limitations that should be acknowledged. First, a high degree of heterogeneity was detected among the included studies, so that a bivariate model was adopted in our meta-analysis to incorporate the uncertainties arising from between study variations. Second, this meta-analysis included a limited number of studies, with only three studies involving 100 participants or more [19, 28, 30]. Given the limited number of larger studies included, more primary research is needed. Third, this meta-analysis was unable to derive an optimal cutoff, as none of the included studies proposed a cutoff value.

## Conclusions

Ultrasound shows excellent diagnostic accuracy for BC as compared with pathology and provides similar diagnostic information (absent or present) compared to MRI. It is therefore recommended as a candidate in the diagnostic examination of patients with BC, especially when MRI is not available or contraindicated.

## Supplementary Information

**Additional file 1.** The complete search strategy and the results of quality assessments.

## Data Availability

The datasets analyzed during the current study are available from the corresponding authors on reasonable request.

## Abbreviations

BC: Baker’s cyst
AUC: Area under the curve
PRISMA: Preferred Reporting Items for Systematic Reviews and Meta-analyses
QUADAS-2: Quality assessment for diagnostic accuracy studies-2
DOR: Diagnostic odds ratio
SROC: Summary receiver operating characteristic
PLR: Positive likelihood ratio
NLR: Negative likelihood ratio
CI: Confidence intervals
