# Supplementary material for: Diagnostic accuracy of ultrasound for the assessment of Baker’s cysts: a meta-analysis
Source: J Orthop Surg Res. 2022 Dec 12;17:535. doi: 10.1186/s13018-022-03430-9 (PMC9743685; doi:10.1186/s13018-022-03430-9)
Supplement: Supplementary file 1 — Additional file 1. The complete search strategy and the results of quality assessments. [file 13018_2022_3430_MOESM1_ESM.docx]

**Supplemental Data 1**

**Search strategy**

**PubMed**:

1. Ultrasonography[mesh] OR ultrasonograph*[tiab] OR sonograph*[tiab] OR ultrasound*[tiab]
2. Popliteal Cyst[mesh] OR Popliteal Cyst*[tiab] OR Cyst, Popliteal[tiab] OR Cysts, Popliteal[tiab] OR Baker's Cyst*[tiab] OR Baker Cyst*[tiab] OR Cyst, Baker*[tiab] OR Cysts, Baker*[tiab]
3. 1 AND 2

**Embase**:

1. 'ultrasound'/exp OR 'ultrasound' OR 'echography'/exp OR 'echography' OR ultrasonograph*:ab,ti OR sonograph*:ab,ti OR ultrasound*:ab,ti
2. 'popliteal cyst'/exp OR 'popliteal cyst*':ti,ab OR 'cyst, popliteal':ti,ab OR 'cysts, popliteal':ti,ab OR 'bakers cyst*':ti,ab OR 'baker cyst*':ti,ab OR 'cyst, baker*':ti,ab OR 'cysts, baker*':ti,ab
3. 1 AND 2

**Web of Science**:

1. ultrasonography (Topic) or ultrasonograph* (Topic) or sonograph* (Topic) or ultrasound* (Topic)
2. Popliteal Cyst* (Topic) or Cyst, Popliteal (Topic) or Cysts, Popliteal (Topic) or Baker's Cyst* (Topic) or Baker Cyst* (Topic) or Cyst, Baker* (Topic) or Cysts, Baker* (Topic)
3. 1 AND 2


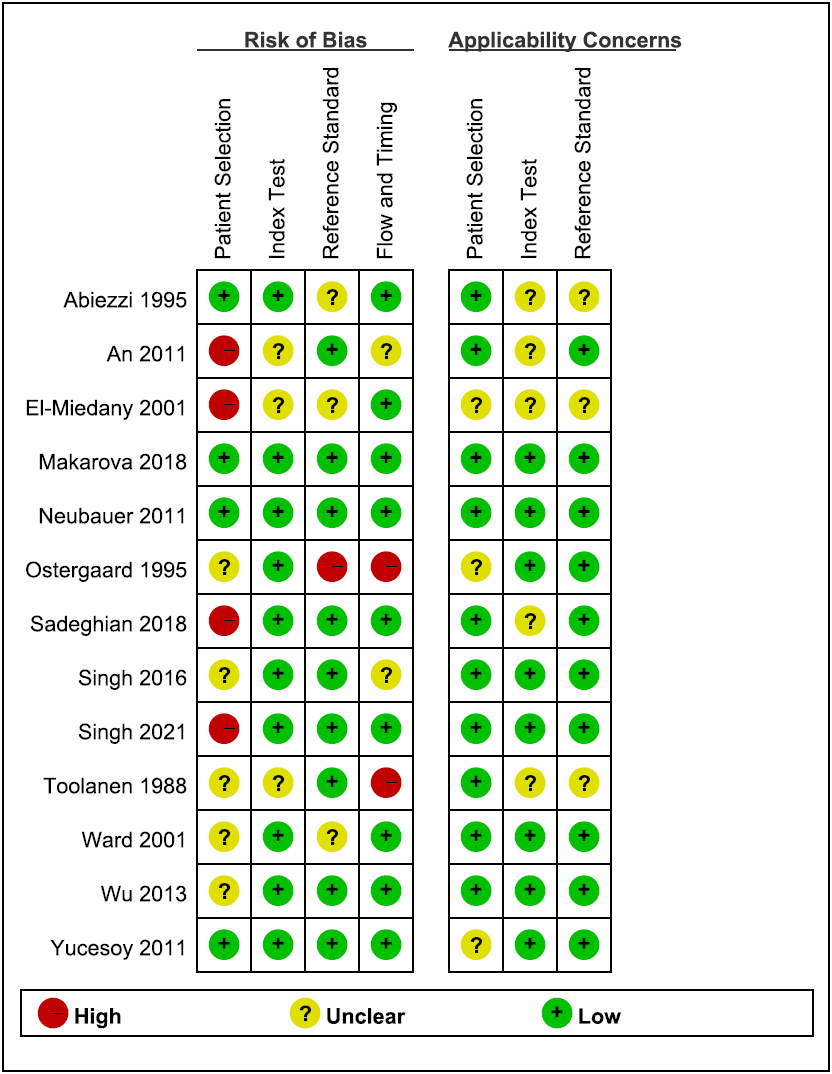


**Supplemental Figure 1. The Cochrane risk of bias and applicability concerns summary.**

**
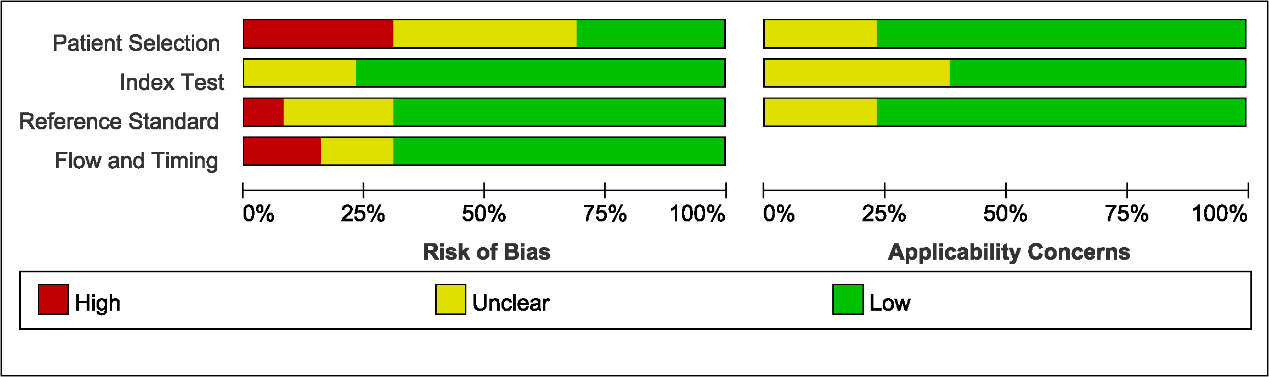
**

**Supplemental Figure 2. The Cochrane risk of bias graph.**
